# *Celastrus orbiculatus* extracts induce apoptosis in mTOR-overexpressed human hepatocellular carcinoma HepG2 cells

**DOI:** 10.1186/s12906-018-2397-0

**Published:** 2018-12-07

**Authors:** Yayun Qian, Ting Yang, Xueyu Zhao, Yan Yan, Wenyuan Li, Chuanci Fang, Jingjing Hou, Li Tao, Yanqing Liu

**Affiliations:** 1 Institute of Traditional Chinese Medicine & Western Medicine, School of Medicine, Yangzhou University, 11 Huaihai Road, Yangzhou, 225009 China; 2 Jiangsu Key Laboratory of Integrated Traditional Chinese and Western Medicine for Prevention and Treatment of Senile Diseases, Yangzhou, 225001 China; 3 Jiangsu Co-innovation Center for Prevention and Control of Important Animal Infectious Diseases and Zoonoses, Yangzhou, 225009 China; 4 Changzhou Hospital of Traditional Chinese Medicine, Changzhou, 213000 China

**Keywords:** Celastrus orbiculatus, Hepatocellular carcinoma, mTOR, Apoptosis

## Abstract

**Background:**

*Celastrus orbiculatus* (Celastraceae) are used as traditional Chinese medicine to treat inflammation and cancer. This study aims to evaluate the effect of *Celastrus orbiculatus* extract (COE) on the apoptosis in human hepatic carcinoma HepG2 cells with mTOR overexpression.

**Methods:**

The stable expression of mTOR in HepG2 cells (HepG2/mTOR^+^) were established by lipofectin transfection of GV238-mTOR recombinant plasmids and further antibiotic selection. Human hepatic carcinoma HepG2/mTOR^+^ cells were treated with different concentrations (20, 40, 80, 160, and 320 μg/mL) of COE for 24 h. The cell proliferation upon COE treatment was detected by MTT. Apoptosis was measured by Flow Cytometry. The activity of mTOR signaling pathway was detected by Western Blotting.

**Results:**

COE significantly inhibited the proliferation of HepG2/mTOR^+^ cells. The expression levels of Bax and Caspase-3 protein were increased in the HepG2/mTOR^+^ cells in a dose-dependent manner. The proteins expression of Bcl2, Bcl-2 L12, mTOR, phospho-mTOR, 4EBP1, phospho-4EBP1, P70S6k, and phospho-P70S6k in HepG2/mTOR^+^ cells were reduced in dose-dependent manners. Furthermore, COE and mTOR inhibitor rapamycin (RAPA) synergistically induced apoptosis in HepG2/mTOR^+^ cells by regulating apoptosis-related proteins and inhibiting mTOR signaling pathways.

**Conclusion:**

COE could inhibit the proliferation of HepG2/mTOR^+^ cells, and induce the cell apoptosis. The mechanisms may be related to the regulation of the expression of Bcl-2, Bcl-2 L12, and mTOR signaling pathways. These data suggest that COE may be a potential treatment for human hepatocellular carcinoma.

## Background

Hepatocellular carcinoma (HCC) is one of the most common malignant tumors in the world [1]. In recent years, the incidence and mortality rate of HCC is increasing. Despite multimodal therapies, including surgery, chemotherapy, and radiotherapy, the curative effect on HCC patients is not as good as anticipated [2]. Recent studies of new anti-metastatic agents have demonstrated that some Chinese herbs with chemopreventive capability can slow down the metastasis of several types of cancer [3, 4]. Previous studies showed that the ethyl acetate extract of *Celastrus orbiculatus* extract (COE) exhibited many significant anti-tumor bioactivities, such as inhibiting proliferation and inducing apoptosis [5–7]. Mechanistic target of rapamycin (mTOR) is associated with poorly differentiated tumors and bad prognosis. The two mTOR-containing complexes (mTORC1 and mTORC2 pathways) that involve pRPS6 and p-AKT are up-regulated by 40–50% in HCCs [8]. Thus, blocking the mTOR signal pathway is an attractive strategy for HCC treatment. Preliminary experimental studies have revealed that COE has a significant inhibitory effect [9–13] on the epithelialmesenchymal transition (EMT), invasion, and metastasis, and inhibits the growth of several types of cancer cells. The preliminary results of our study suggest that COE can inhibit the activity of the mTOR signaling pathway [14], but the underlying molecular mechanism has not been revealed completely. This study explored the effects of COE on the proliferation and apoptosis in the HepG2/mTOR^+^ cells, which may bring new hope for clinical treatment of cancer characterized with mTOR activation.

## Materials and methods

### Preparation of extract

The dried stems of the *C. orbiculatus* were provided by Zhixin Pharmaceutical Co., Ltd. (Guangzhou, China). As described previously [5, 9–14], the authentication and preparation of COE was made by professor Wangqiang (China Pharmaceutical University) [15]. Briefly, the powder of the herb was extracted with 10-fold of 95% ethanol under heat for 3 h three times and the mixtures were filtered and concentrated. Then the obtained extractions from ethyl acetate were concentrated using a rotary evaporator and stored at − 20 °C. Before use, the extracts were dissolved in DMSO with the final concentration of DMSO not exceeding 0.1%. The positive control drug, Cisplatin (abbreviated to DDP, 2 mg/L), was product of Haosen Pharmaceutical Co., Ltd. (Jiangsu, China) [16].

### Chemical reagents and antibodies

DMEM and fetal bovine serum (FBS) was obtained from GIBCO-BRL (Gaithersburg, MD, USA). The antibodies, including rabbit β-actin, mTOR, phospho-mTOR, 4E-BP1, phospho-4E-BP1, P70S6k, and phospho-P70S6k were purchased from Cell Signaling Technology (Beverly, MA). Rabbit Bax antibody was acquired from Santa Cruz in USA. Rabbit Bcl-2, Bcl-2 L12, and Caspase-3 antibody from American Epitomics Company were also obtained. HRP labeled goat anti-rabbit IgG was purchased from Hangzhou Huaan Biotechnology Co.

### Cell culture

Human hepatocellular carcinoma HepG2 cells were obtained from the Cell Bank of Chinese Academy of Sciences Shanghai Institute of Cell Biology (Shanghai, China). The HepG2 Cells with high expression of mTOR, termed as HepG2/mTOR^+^, were constructed by our laboratory. The cells were cultured in DMEM which was supplemented with 10% FBS at 37 °C in a humidified incubator containing 5% CO_2_.

### Cell viability assay

The cell viability was determined using MTT assay. HepG2/mTOR^+^ were inoculated at a density of 1 × 10^4^ cells per well in 96-well plates, treated with COE at various concentrations (20, 40, 80, 160 and 320 μg/mL). The cell incubated only DMSO was considered as the negative control. The incubation was continued for 24, 48, and 72 h, respectively. Subsequently, 20 μL of MTT was added to plates and incubated for another 4 h. The supernatant was gently discarded and replaced with 150 μL DMSO to dissolve the formazan crystal. The absorbance (A) value was detected at 490 nm. Each experiment was repeated for three times.

### Flow cytometry

HepG2/mTOR^+^ Cells treated with different concentrations of COE for 24 h, cells were washed with PBS by centrifugation for 5 min. Subsequently, cells were incubated with 5 μL Annexin V-FITC and 5 μL PI or FITC isotype control for 30 min at 4 °C in the dark. The levels of fluorescence were analyzed with FACSort software (Becton-Dickinson, USA). Each assay was performed with three independent experiments.

### Western blot analysis

HepG2/mTOR^+^ Cells were incubated with different concentrations of COE for 24 h. The total proteins, extracted with cell lysis buffer (Beyotime, Jiangsu, China) for 30 min on ice, were quantified by NanoPhotometer pearl (IMPLEN, Germany). 50 μg of total protein were separated on 10% SDS-PAGE for electrophoresis, and then transferred to PVDF membranes. The membranes were blocked with 5% BSA for 2 h, and then incubated with appropriate primary antibodies overnight at 4 °C. The following day, the membranes were incubated with the secondary antibody for 2 h and detected by using the ECL reagent.

### Statistical analysis

All experiments were performed in triplicate, and the results are presented as mean ± standard deviation. Statistical analysis was carried out with GraphPad Prism 5.0 Software. The unpaired Student’s *t*-test was used to determine *P*-values for the differences. Results were considered significantly different when *P* < 0.05.

## Results

### Establishment of the stable HepG2 cell line with mTOR overexpression

**Fig. 1:**
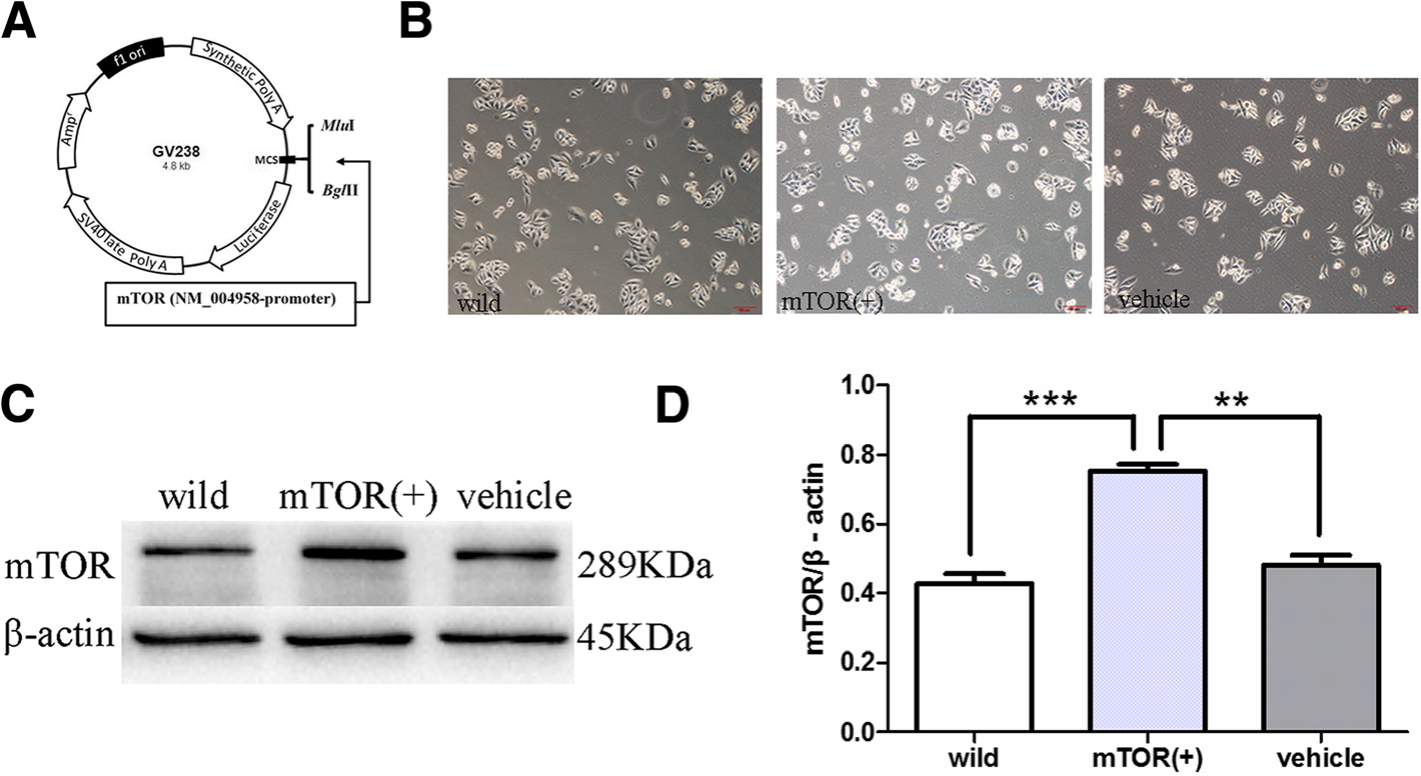
Generation of the HepG2/mTOR^+^ cells. **a** Construction of the GV238-mTOR recombinant plasmid. **b** cell morphology under a microscope (100×). **c** and **d** the expression levels of mTOR in the HepG2/mTOR^+^ cells. (^**^P < 0.01, versus vehicle; ***P < 0.001, versus wild type)

The GV238 vector with Luciferase reporter gene was digested with *Mlu*I and *Bgl*II. And then the mTOR (NM_004958) promotor genes were cloned into GV238 vector by using molecular biological technology (Fig. 1a). The GV238-mTOR recombinant plasmids were transfected into HepG2 cells, named as HepG2/mTOR^+^ cells, with using transfected GV238 vehicle HepG2 cells as control [17]. HepG2/mTOR^+^ cells in logarithmic growth phase were seeded in a 6-well plate at 5 × 10^4^ /well. The cell morphology has no appreciable difference after mTOR gene transfection under a microscope (100× magnification) (Fig. 1b). The mTOR protein expression was detected by western blots. Compared to the wild type HepG2 cells, the mTOR protein expression in HepG2/mTOR^+^ cells were significantly increased (Fig. 1c, d). These results showed that we had successfully established a stable HepG2 cell line with overexpressed mTOR.

### COE inhibited the viability in the HepG2/ mTOR^+^ cells

**Fig. 2:**
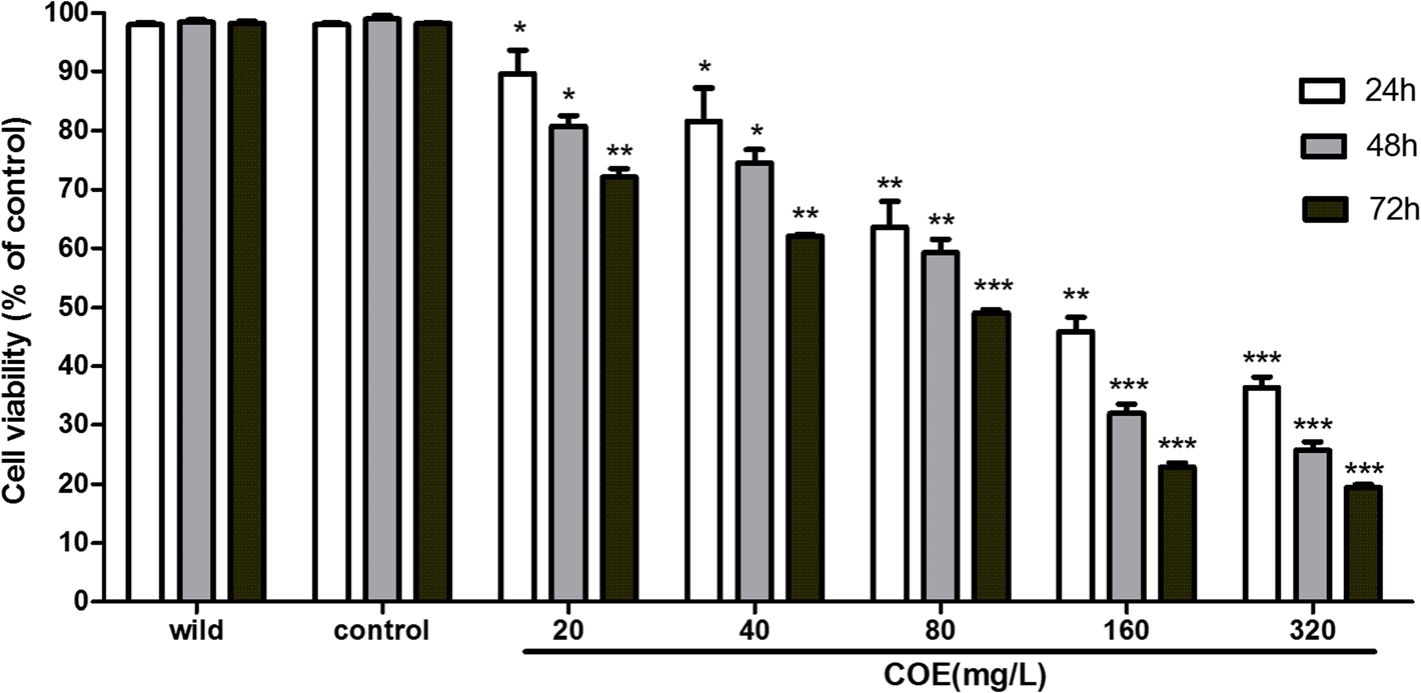
Effects of COE on the viability of the HCC cells. The HepG2/mTOR^+^ cells were treated with either 0.1%DMSO as solvent control or different concentrations of COE (20, 40, 80, 160, and 320 mg/L) for 24, 48, and 72 h, respectively, and the cell viability was evaluated with MTT assay. (^*^P < 0.05, ^**^P < 0.01, ^***^P < 0.001, compared with the solvent control)

After adding different concentrations of COE (20, 40, 80, 160, and 320 mg/L), the cell viability was investigated by MTT for 24, 48, and 72 h, respectively. Compared with the vehicle and wide type HepG2 cells, the growth of untreated HepG2/mTOR^+^ cells has not shown any significant difference. On the other hand, in the treated groups, the growth of HepG2/mTOR^+^ cells was inhibited significantly in a dose-dependent and time-dependent manner (Fig. 2). The half inhibitory concentration of COE for 24 h was 126 mg/L. In order to decrease the cytotoxicity of the drug, the concentrations (20, 40, and 80 mg/L) of COE were selected for further study.

### Morphology of the HepG2/ mTOR^+^ cells

**Fig. 3:**
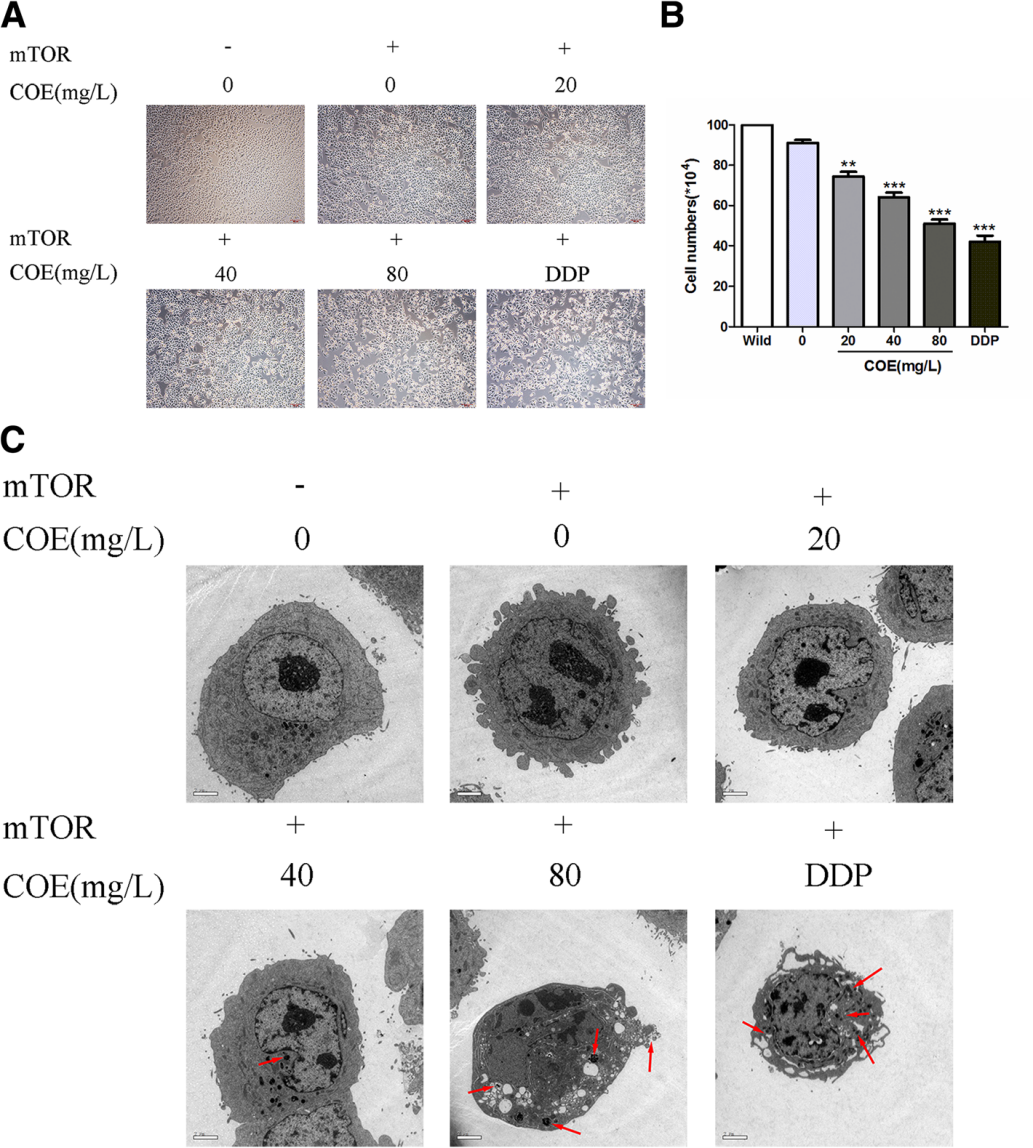
Effects of COE on cellular morphology of the HCC cells. The HepG2/mTOR^+^ cells were treated with 0.1%DMSO as solvent control, or 2 mg/L DDP, or different concentrations of COE (20, 40, and 80 mg/L) for 24 h. **a** and **b** the HepG2/mTOR^+^ cells were observed under inverted microscope and taken phase-contrast images from the same fields (100×). **c** the morphological changes were observed under transmission electron microscope (2950×, Scale, 1 μm); red arrows are representative of the apoptotic bodies. (^**^P < 0.01, ^***^P < 0.001, compared with the vehicle)

Phase-contrast images of cells from the same fields were taken 24 h after the treatment of COE. Representative pictures of HepG2/mTOR^+^ cells showed that the viability was significantly decreased (Fig. 3a, b). Transmission electron microscopy demonstrated that there were many microvilli and fenestrations on the cellular surface of the wide type HepG2 cells. There were many organelles in the cytoplasm, and the mitochondria were regular. After the treatment of COE, the cytoplasm was concentrated, the cell membrane was bubbled, and the apoptotic bodies were produced (Fig. 3c). It was shown that some cells were necrotic, and the cell membranes ruptured with the contents were released.

### COE induced the apoptosis in HepG2/ mTOR^+^ cells

**Fig. 4:**
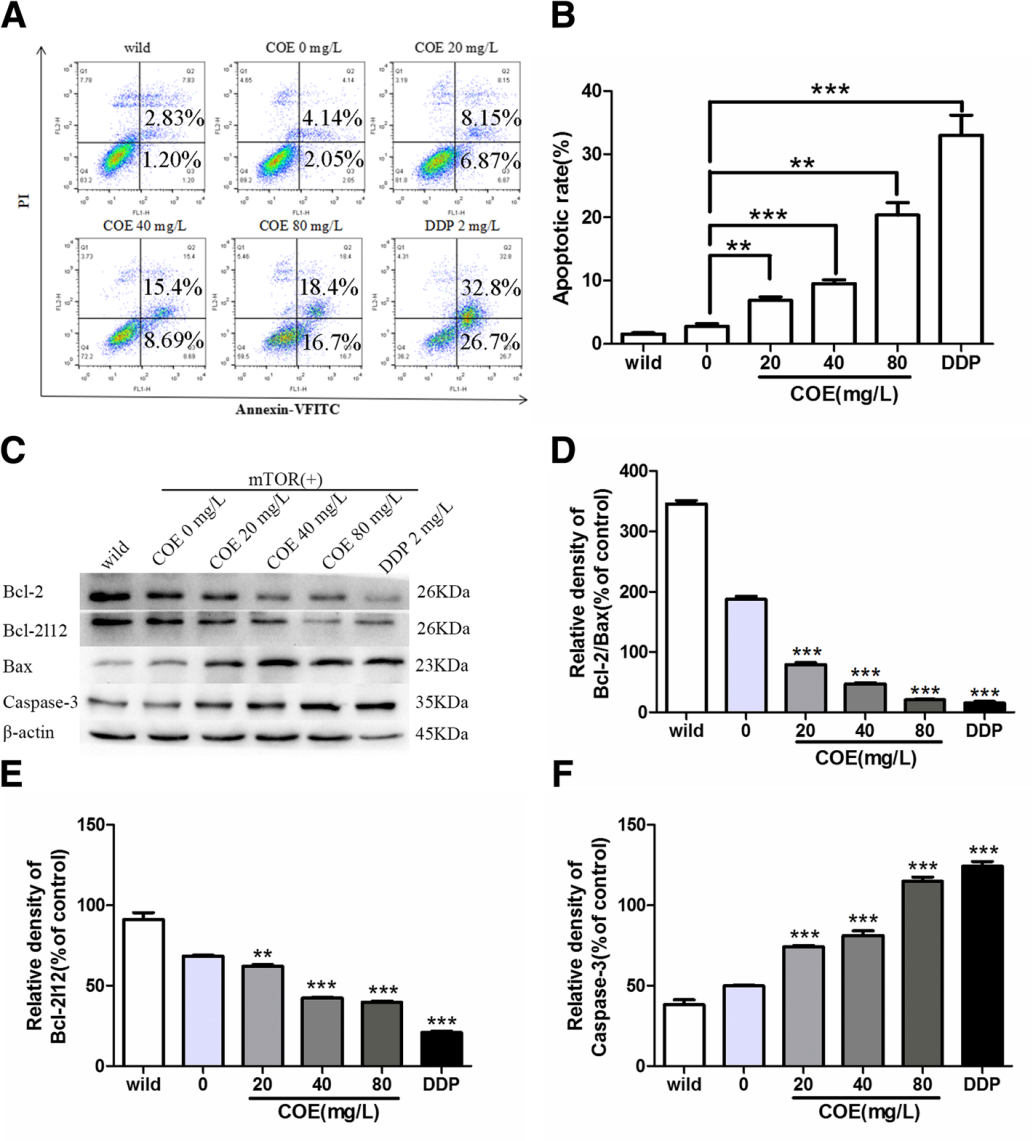
COE promoted apoptosis of the HCC cells. The HepG2/mTOR^+^ cells were treated with 0.1%DMSO as solvent control, or 2 mg/L DDP, or different concentrations of COE (20, 40, 80 mg/L) for 24 h. **a** and **b** the apoptosis was detected by Flow cytometry. **c**-**f** the protein expression of Bcl-2, Bcl-2 L12, Bax, and Caspase-3 were examined by Western blots. (^**^P < 0.01, ^***^P < 0.001, compared with the vehicle)

After adding different concentrations of COE (20, 40, and 80 mg/L) for 24 h, apoptosis was detected by flow cytometric analysis (2 mg/L DDP was used as the positive control drug). There was no significant difference between the wild type HepG2 cells and HepG2/mTOR^+^ cells. And the results showed that after the drug treatment, the percentage of the apoptotic HepG2/mTOR^+^ cells was significantly increased in a dose-dependent manner (Fig. 4a, b). The results of Western blots showed that COE increased the expression of Bax and Caspase-3. Meanwhile, COE decreased the expression of Bcl-2 and Bcl-2 L12 in a concentration-dependent manner, especially reducing the ratio of Bcl-2/Bax (Fig. 4c-e). It indicated that COE induced the apoptosis of the HepG2/mTOR^+^ cells in a concentration-dependent manner.

### COE effects on the mTOR signaling pathway

**Fig. 5:**
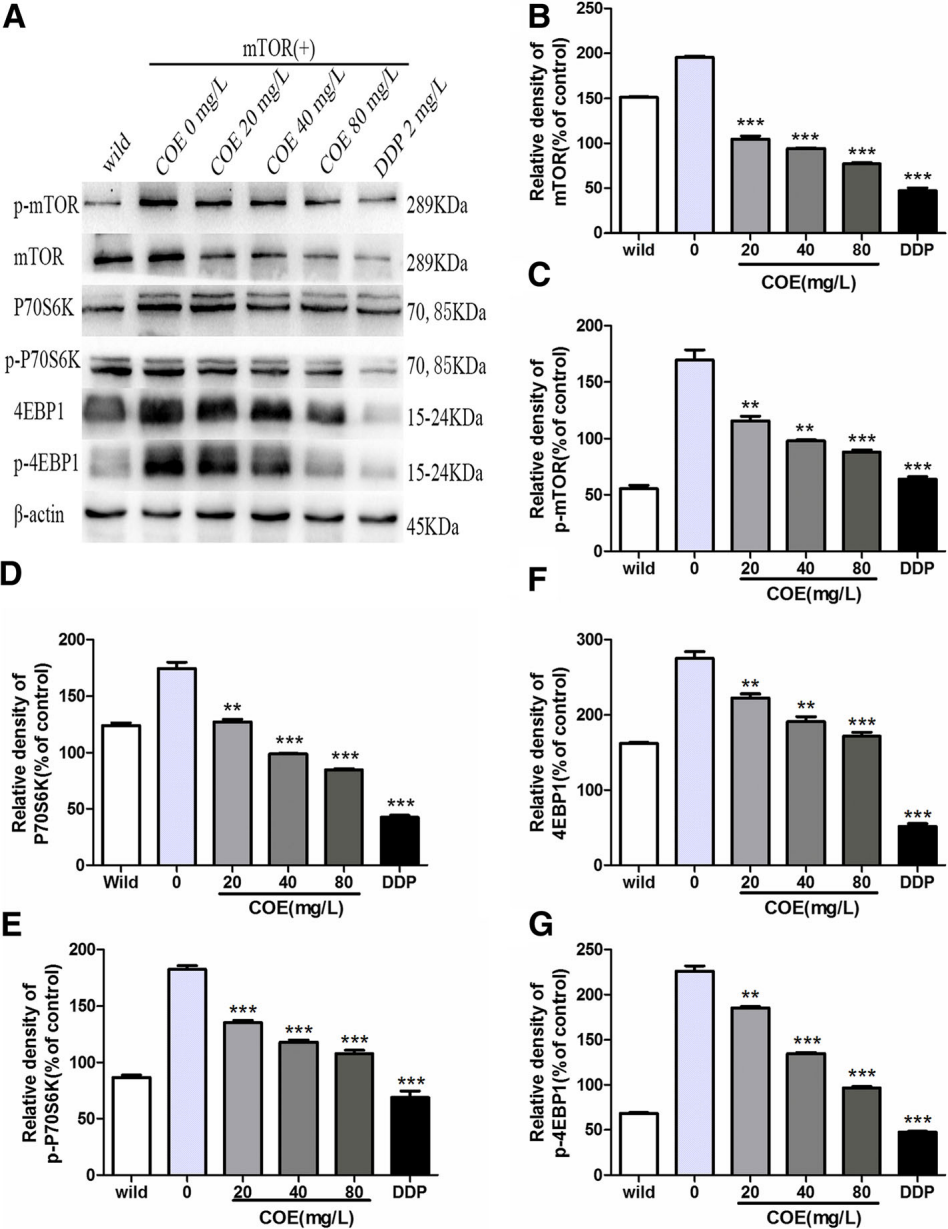
Expression of the proteins that are involved in the mTOR signaling pathways. The HepG2/mTOR^+^ cells were treated with 0.1%DMSO as solvent control, or 2 mg/L DDP, or different concentrations of COE (20, 40, and 80 mg/L) for 24 h. The proteins expression of mTOR, p-mTOR, 4E-BP1, p-4E-BP1, P70S6K, and p-P70S6K were studied by Western blot analysis. (**P < 0.01, ***P < 0.001, compared with the vehicle control)

After adding different concentrations of COE (20, 40, and 80 mg/L) for 24 h, the protein expression correlated mTOR signaling pathways were determined by Western blots (2 mg/L DDP was used as the positive control drug). Compared to the untreated control, in HepG2/mTOR^+^ cells, the protein levels of mTOR, p-mTOR and its downstream proteins such as 4EBP1, p-4EBP1, P70S6k, and p-P70S6k, were reduced significantly in both dose-dependent and time-dependent manners after COE treatment (Fig. 5).

**Fig. 6:**
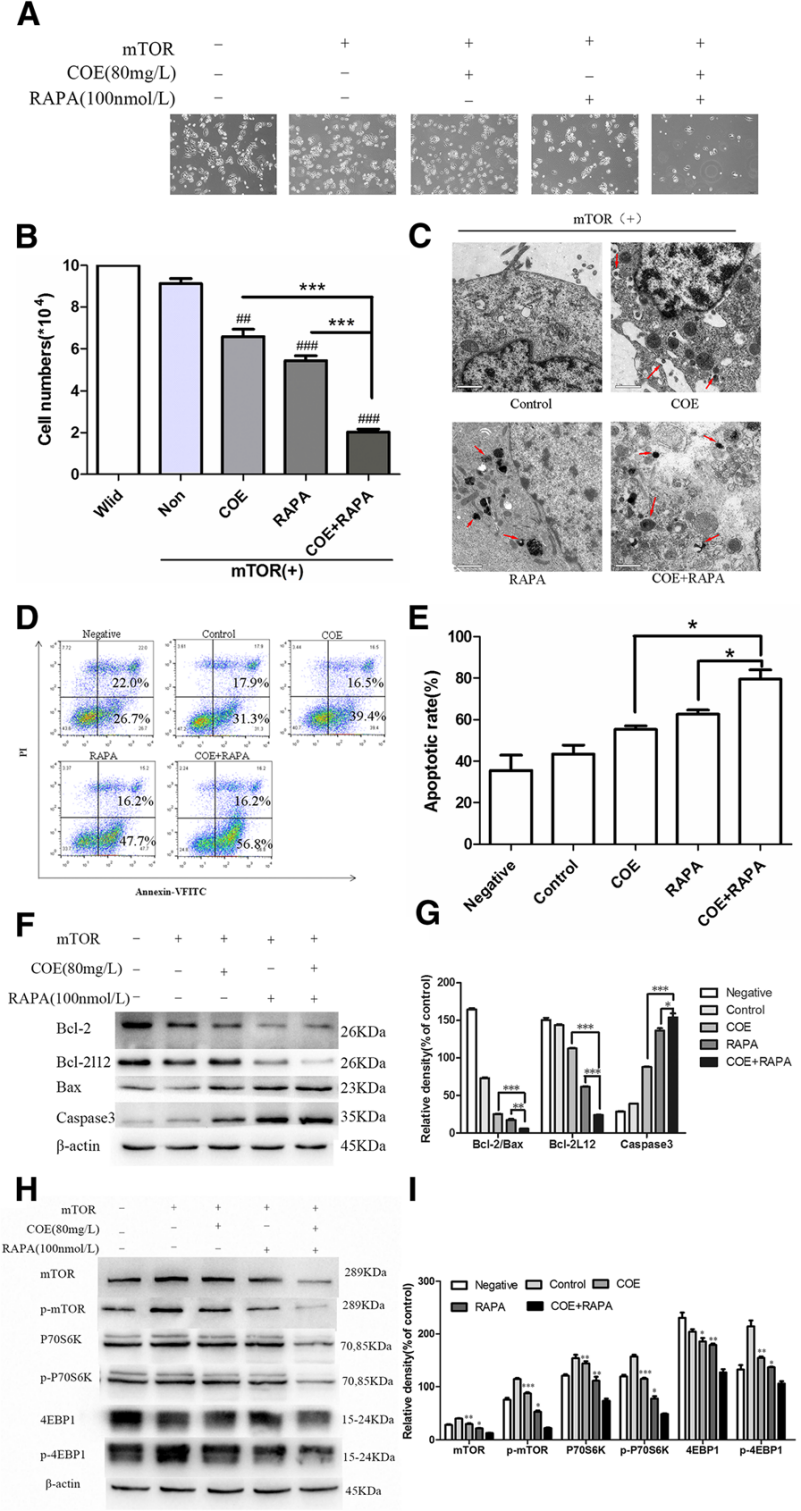
Effects of the combined treatment of RAPA and COE. The HepG2/mTOR^+^ cells were treated with 0.1%DMSO as solvent control, or 80 mg/L COE, or 100 nmol/L RAPA combined with 80 mg/L COE for 24 h. **a** and **b** the numbers of the HepG2/mTOR^+^ cells were observed under inverted microscope (100×). **c** the morphological changes were observed under transmission electron microscope (2950×, Scale, 1 μm); red arrows are representative of the apoptotic bodies. **d** and **e** the apoptosis was detected by Flow cytometry. **f** and **g** the proteins expression of Bcl-2, Bcl-2 L12, Bax, and Caspase-3 were examined by Western blots. **h** and **i** the proteins expression of mTOR, p-mTOR, 4E-BP1, p-4E-BP1, P70S6K, and p-P70S6K were accessed by Western blots. (^*^P < 0.05, ^**^P < 0.01, ^***^P < 0.001, COE + RAPA group compared with COE or RAPA alone; ^##^*P* < 0.01, ^###^*P* < 0.001, compared to the control)

The mTOR signaling pathway is a master regulator of cell growth and metabolism. Dysregulation of the mTOR pathway has been implicated in a number of human diseases such as cancer, diabetes, obesity, neurological diseases, and genetic disorders. Rapamycin (RAPA), a specific inhibitor of mTOR, has been shown to be effective in treating several diseases [18]. In order to confirm whether COE has a synergistic effect with mTOR inhibitors and induces apoptosis via mTOR signaling pathways in HepG2/mTOR^+^ cells, we used 100 nmol/L RAPA to observe the effects of COE on apoptosis. The results showed that COE reduced the cell number (Fig. 6a, b) and induced apoptosis (Fig. 6d-g) in HepG2/mTOR^+^ cells. The cell morphology was observed by transmission electron microscopy after the co-treatment of RAPA and COE. The cell membranes were ruptured and the contents were released (Fig. 6c). The expression levels of the proteins that are involved in mTOR signaling pathways were changed significantly. Compared with the treatment with COE or RAPA alone, the co-treatment of COE and RAPA showed a synergistic effect in HepG2/mTOR^+^ cells (Fig. 6h, i). Taken together, these data revealed that COE could further promote tumor cell apoptosis when mTOR signaling pathways are suppressed.

## Discussion

Many extracts derived from herbs have been tested as inhibitors of cancer cell proliferation both in vitro and in vivo [19–21]. The preliminary results of our study have demonstrated that COE is cytotoxic to various cancer cells including human glioblastoma cells [11], hepatocellular carcinoma [5–7], and human gastric cancer [9, 10, 12, 13]. Mammalian target of rapamycin (mTOR) is a class of non-conserved evolutionary protein kinase, and involved in a variety of physiological and pathological processes, such as cell proliferation, cell differentiation, autophagy, angiogenesis, *etc* [22–25]. The two mTOR-containing complexes (mTORC1 and mTORC2) have different sensitivities to rapamycin. mTORC1 is inhibited by a complex consisted of rapamycin and FKBP12 protein [26]. In contrast, mTORC2 is generally resistant to rapamycin, however, in certain cell types, mTORC2 may show sensitivity after prolonged rapamycin treatment [27]. Accumulated evidence supports that there are mutations, amplifications, or deletions of mTOR signaling pathways in many tumors. These proteins can cause over-activation of mTOR pathways, leading to abnormal tumor cell proliferation [28]. Clinical specimens from patients with hepatocellular carcinoma were analyzed by using immunohistochemistry [29]. The results showed that the expression level of mTOR is higher than that in the adjacent non-tumor liver tissue, and protein expression level of mTOR was positively correlated with malignancy and poor prognosis. This suggests that mTOR may be a potential target for the treatment of hepatocellular carcinoma. Biomarkers for mTOR inhibitor efficacy have been evaluated in both preclinical and clinical studies. Our data identified that COE is able to inhibit mTOR signaling pathways.

The Bcl-2 family is the key factor in the mitochondria-mediated signal pathway of apoptosis [30]. Bcl-2 is an inhibitor of apoptosis, preventing the release of mitochondrial cytochrome c, while Bax is a pro-apoptotic factor that in turn promotes its release. Bcl-2 L12 has been discovered as a new gene of Bcl-2 family which can inhibit apoptosis of tumor cell [31, 32] and was found to be over-expressed in tumor tissue [33]. Caspase-3 is another important terminal cleaving enzyme in the process of cell apoptosis [34]. This study indicated that COE could reduce the expression of Bcl-2 protein and increase the expression of Bax and Casepase-3 total protein, while the ratio of Bcl-2/Bax was decreased. Therefore, COE played a pro-apoptotic role through the Bcl-2, Bax, and Casepase-3-mediated signaling pathway. The results of the present study demonstrated that COE inhibited the proliferation of HepG2/mTOR^+^ cells and induced apoptosis in a concentration-dependent manner. Furthermore, the combination of COE and RAPA synergistically induced apoptosis in HCC cells by regulating apoptosis-related proteins and inhibiting the mTOR signaling pathways.

## Conclusion

In summary, COE contributed to promote apoptosis of HepG2/mTOR^+^ cells, which was closely related to Bcl-2 family. Also, COE was able to suppress the mTOR signaling pathways. Nevertheless, in vivo data are still required for further verifying our findings. Altogether, the present study reveals that COE can be considered as a potential antineoplastic drug for treating hepatocellular carcinoma.

## Abbreviations

COE: *Celastrus orbiculatus* extract
HCC: Hepatocellular carcinoma
RAPA: Rapamycin
